# Supplementary material for: The Top 50 Most Cited Articles on Special Olympics: A Bibliometric Analysis
Source: Int J Environ Res Public Health. 2022 Aug 16;19(16):10150. doi: 10.3390/ijerph191610150 (PMC9407739; doi:10.3390/ijerph191610150)
Supplement: Supplementary file 1 [file ijerph-19-10150-s001.zip › ijerph-1842043-supplementary.pdf]

The Top 50 Highly Cited Special Olympics Publications Based on Total Citations Between 1978 and 4 March 2022

Publication	Subject area	TC	ACPY
Dykens, E.M.; Cohen, D.J. Effects of Special Olympics International on social competence in persons with mental retardation. *Journal of the American Academy of Child & Adolescent Psychiatry* **1996**, *35*, 223-229.	Psycho-social health	71	2.73
Weiss, J.; Diamond, T.; Demark, J.; Lovald, B. Involvement in Special Olympics and its relations to self-concept and actual competency in participants with developmental disabilities. *Research in Developmental Disability* **2003**, *24*, 281-305.	Psycho-social health	69	3.63
Özer, D.; Baran, F.; Aktop, A.; Nalbant, S.; Ağlamış, E.; Hutzler, Y. Effects of a Special Olympics Unified Sports soccer program on psycho-social attributes of youth with and without intellectual disability. *Research in Developmental Disability* **2012**, *33*, 229-239.	Psycho-social health	65	6.50
Pezzementi, M.L.; Fisher, M.A. Oral health status of people with intellectual disabilities in the southeastern United States. *The Journal of the American Dental Association* **2005**, *136*, 903-912.	Oral health	65	3.82
Dykens, E.M.; Rosner, B.A.; Butterbaugh, G. Exercise and sports in children and adolescents with developmental disabilities: positive physical and psychosocial effects. *Child and Adolescent Psychiatric Clinics of North America* **1998**, *7*, 757-771.	Physical and psychosocial health	65	2.71
McConkey, R.; Dowling, S.; Hassan, D.; Menke, S. Promoting social inclusion through unified sports for youth with intellectual disabilities: a five-nation study. *Journal of Intellectual Disability Research* **2013**, *57*, 923-935.	Social inclusion	60	6.67
Farrell, R.J.; Crocker, P.R.; McDonough, M.H.; Sedgwick, W.A. The driving force: motivation in Special Olympians. *Adapted Physical Activity Quarterly* **2004**, *21*, 153-166.	Psycho-social health	52	2.89
Feldman, C.A.; Giniger, M.; Sanders, M.; Saporito, R.; Zohn, H.K.; Perlman, S.P. Special Olympics, special smiles: Assessing the feasibility of epidemiologic data collection. *The Journal of the American Dental Association* **1997**, *128*, 1687-1696.	Oral health	52	2.08
Harada, C.M.; Siperstein, G.N. The sport experience of athletes with intellectual disabilities: A national survey of Special Olympics athletes and their families. *Adapted Physical Activity Quarterly* **2009**, *26*, 68-85.	Motives for participating in Special Olympics	49	3.77
Harris, N.; Rosenberg, A.; Jangda, S.; O'Brien, K.; Gallagher, M.L. Prevalence of obesity in International Special Olympic athletes as determined by body mass index. *Journal of the American Dietetic Association* **2003**, *103*, 235-237.	BMI & Risk of obesity	48	2.53
Khoo, S.; Engelhorn, R. Volunteer motivations at a national Special Olympics event. *Adapted Physical Activity Quarterly* **2011**, *28*, 27-39.	Retaining volunteers/volunteer motivation	44	4.00
Gibbons, S.L.; Byshakra, F.B. Effects of Special Olympics participation on the perceived competence and social acceptance of mentally retarded children. *Adapted Physical Activity Quarterly* **1989**, *6*, 40-51.	Psychosocial health	44	1.33

Castagno, K.S. Special Olympics unified sports: Changes in male athletes during a basketball season. *Adapted Physical Activity Quarterly* **2001**, *18*, 193-206.	Psycho-social health	41	1.95
Reid, B.C.; Chenette, R.; Macek, M.D. Prevalence and predictors of untreated caries and oral pain among Special Olympic athletes. *Special Care in Dentistry* **2003**, *23*, 139-142.	Oral health	40	2.11
Riggen, K.; Ulrich, D. The effects of sport participation on individuals with mental retardation. *Adapted Physical Activity Quarterly* **1993**, *10*, 42-51.	Psycho-social health	40	1.38
Wu, Y.D.; Li, C.X.; Khoo, S. Predicting Future Volunteering Intentions Through a Self-determination Theory Perspective. *Voluntas* **2016**, *27*, 1266-1279, doi:10.1007/s11266-015-9570-6.	Retaining volunteers/volunteer motivation	39	6.50
Turner, S.; Sweeney, M.; Kennedy, C.; Macpherson, L. The oral health of people with intellectual disability participating in the UK Special Olympics. *Journal of Intellectual Disability Research* **2008**, *52*, 29-36.	Oral health	38	2.71
Pitetti, K.H.; Jackson, J.A.; Stubbs, N.B.; Campbell, K.D.; Battar, S.S. Fitness levels of adult Special Olympic participants. *Adapted Physical Activity Quarterly* **1989**, *6*, 354-370.	Physical fitness	38	1.15
Kim, M.; Trail, G.T.; Lim, J.; Kim, Y.K. The role of psychological contract in intention to continue volunteering. *Journal of Sport Management* **2009**, *23*, 549-573.	Retaining volunteers/volunteer motivation	37	2.85
Woodhouse, J.M.; Adler, P.; Duignan, A. Vision in athletes with intellectual disabilities: the need for improved eyecare. *Journal of Intellectual Disability Research* **2004**, *48*, 736-745.	Eye health	37	2.06
Balic, M.G.; Mateos, E.C.; Blasco, C.G.; Fernhall, B. Physical fitness levels of physically active and sedentary adults with Down syndrome. *Adapted Physical Activity Quarterly* **2000**, *17*, 310-321.	Physical fitness	36	1.64
Harada, C.M.; Siperstein, G.N.; Parker, R.C.; Lenox, D. Promoting social inclusion for people with intellectual disabilities through sport: Special Olympics International, global sport initiatives and strategies. In *Disability in the global sport arena*; Routledge: 2013; pp. 73-90.	Social inclusion	35	3.18
Cuesta-Vargas, A.I.; Paz-Lourido, B.; Rodriguez, A. Physical fitness profile in adults with intellectual disabilities: differences between levels of sport practice. *Research in Developmental Disabilities* **2011**, *32*, 788-794.	Physical fitness	34	3.09
Hild, U.; Hey, C.; Baumann, U.; Montgomery, J.; Euler, H.; Neumann, K. High prevalence of hearing disorders at the Special Olympics indicate need to screen persons with intellectual disability. *Journal of Intellectual Disability Research* **2008**, *52*, 520-528.	Hearing health	33	2.36
Lloyd, M.; Temple, V.A.; Foley, J.T. International BMI comparison of children and youth with intellectual disabilities participating in Special Olympics. *Research in Developmental Disabilities* **2012**, *33*, 1708-1714.	BMI & risk of obesity	32	3.20
Temple, V.; Foley, J.; Lloyd, M. Body mass index of adults with intellectual disability participating in Special Olympics by world region. *Journal of Intellectual Disability Research* **2014**, *58*, 277-284.	BMI & Risk of obesity	31	3.88

Nasuti, G.; Stuart-Hill, L.; Temple, V.A. The six-minute walk test for adults with intellectual disability: A study of validity and reliability. *Journal of Intellectual and Developmental Disability* **2013**, *38*, 31-38.	Physical fitness	31	3.44
Dellavia, C.; Pallavera, A.; Orlando, F.; Sforza, C. Postural stability of athletes in Special Olympics. *Perceptual and Motor Skills* **2009**, *108*, 608-622.	Spine health	31	2.38
Klein, T.; Gilman, E.; Zigler, E. Special Olympics: An evaluation by professionals and parents. *Mental Retardation* **1993**, *31*, 15-23.	Attitudes towards intellectual disability	30	1.03
Burns, M.; Storey, K.; Certo, N.J. Effect of service learning on attitudes towards students with severe disabilities. *Education and Training in Mental Retardation and Developmental Disabilities* **1999**, 58-65.	Attitudes towards intellectual disability	29	1.26
Gençöz, F. The effects of basketball training on the maladaptive behaviors of trainable mentally retarded children. *Research in Developmental Disabilities* **1997**, *18*, 1-10.	Improved behavior	28	1.12
Ward, L.M.; Cooper, S.A.; Hughes-McCormack, L.; Macpherson, L.; Kinnear, D. Oral health of adults with intellectual disabilities: a systematic review. *Journal of Intellectual Disability Research* **2019**, *63*, 1359-1378, doi:10.1111/jir.12632.	Oral health	27	9.00
Shapiro, D.R. Participation motives of Special Olympics athletes. *Adapted Physical Activity Quarterly* **2003**, *20*, 150-165.	Psychosocial health	27	1.42
Tint, A.; Thomson, K.; Weiss, J.A. A systematic literature review of the physical and psychosocial correlates of Special Olympics participation among individuals with intellectual disability. *Journal of Intellectual Disability Research* **2017**, *61*, 301-324, doi:10.1111/jir.12295.	Physical and psychosocial health	26	5.20
Hsieh, K.; Hilgenkamp, T.I.; Murthy, S.; Heller, T.; Rimmer, J.H. Low levels of physical activity and sedentary behavior in adults with intellectual disabilities. *International Journal of Environmental Research and Public Health* **2017**, *14*, 1503.	Physical activity and sedentary behaviour	26	5.20
Foley, J.T.; Lloyd, M.; Vogl, D.; Temple, V.A. Obesity trends of 8–18 year old Special Olympians: 2005–2010. *Research in Developmental Disabilities* **2014**, *35*, 705-710.	BMI & Risk of obesity	26	3.25
Dellavia, C.; Allievi, C.; Pallavera, A.; Rosati, R.; Sforza, C. Oral health conditions in Italian Special Olympics athletes. *Special Care in Dentistry* **2009**, *29*, 69-74.	Oral health	25	1.92
Storey, K. The more things change, the more they are the same: Continuing concerns with the Special Olympics. *Research and Practice for Persons with Severe Disabilities* **2008**, *33*, 134-142.	Pros and cons of Special Olympics	25	1.79
Roper, P. Changing perceptions through contact. *Disability, Handicap & Society* **1990**, *5*, 243-255.	Attitudes towards intellectual disability	25	0.78
Cuesta-Vargas, A.; Hilgenkamp, T. Reference values of grip strength measured with a Jamar dynamometer in 1526 adults with intellectual disabilities and compared to adults without intellectual disability. *PLoS One* **2015**, *10*, e0129585.	Physical fitness	24	3.43

Baran, F.; Aktop, A.; Özer, D.; Nalbant, S.; Ağlamış, E.; Barak, S.; Hutzler, Y. The effects of a Special Olympics Unified Sports Soccer training program on anthropometry, physical fitness and skilled performance in Special Olympics soccer athletes and non-disabled partners. *Research in Developmental Disabilities* **2013**, *34*, 695-709.	Physical fitness	23	2.56
Storey, K. The case against the Special Olympics. *Journal of Disability Policy Studies* **2004**, *15*, 35-42.	Pros and cons of Special Olympics	23	1.28
Woodhouse, J.M.; Adler, P.M.; Duignan, A. Ocular and visual defects amongst people with intellectual disabilities participating in Special Olympics. *Ophthalmic and Physiological Optics* **2003**, *23*, 221-232.	Eye health	23	1.21
Fernandez, J.B.; Lim, L.J.; Dougherty, N.; LaSasso, J.; Atar, M.; Daronch, M. Oral health findings in athletes with intellectual disabilities at the NYC Special Olympics. *Special Care in Dentistry* **2012**, *32*, 205-209.	Oral health	22	2.20
Bissar, A.R.; Kaschke, I.; Schulte, A.G. Oral health in 12-to 17-year-old athletes participating in the German Special Olympics. *International Journal of Paediatric Dentistry* **2010**, *20*, 451-457.	Oral health	22	1.83
Neumann, K.; Dettmer, G.; Euler, H.A.; Giebel, A.; Gross, M.; Herer, G.; Hoth, S.; Lattermann, C.; Montgomery, J. Auditory status of persons with intellectual disability at the German Special Olympic Games. *International Journal of Audiology* **2006**, *45*, 83-90.	Hearing health	22	1.38
Platt, L.S. Medical and orthopaedic conditions in Special Olympics athletes. *Journal of Athletic Training* **2001**, *36*, 74-80.	General health of people with intellectual disability	22	1.05
Block, S.S.; Beckerman, S.A.; Berman, P.E. Vision profile of the athletes of the 1995 Special Olympics World Summer Games. *Journal of the American Optometric Association* **1997**, *68*, 699-708.	Eye health	22	0.88
McNamara, P.; Pazzaglia, F.; Sonpar, K. Large-scale events as catalysts for creating mutual dependence between social ventures and resource providers. *Journal of Management* **2018**, *44*, 470-500.	Resource mobilization	21	5.25
Goodwin, D.L.; Fitzpatrick, D.A.; Thurmeier, R.; Hall, C. The decision to join Special Olympics: Parents' perspectives. *Adapted Physical Activity Quarterly* **2006**, *23*.	Motives for participating in Special Olympics	21	1.31

TC: Total citations; ACPY: Average citations per year.
